# Management of Odontogenic Sinusitis: Results with Single-Step FESS and Dentoalveolar Surgery

**DOI:** 10.3390/jpm13091291

**Published:** 2023-08-23

**Authors:** Anna Rangics, Gábor Dénes Répássy, Szabolcs Gyulai-Gaál, Csaba Dobó-Nagy, László Tamás, László Simonffy

**Affiliations:** 1 Department of Oral Diagnostics, Faculty of Dentistry, Semmelweis University, 1085 Budapest, Hungary; anna.rangics@gmail.com (A.R.); gyulai@fok.usn.hu (S.G.-G.); dobo@fok.usn.hu (C.D.-N.); laszlo.simonffy@dent.semmelweis-univ.hu (L.S.); 2 Department of Otorhinolaryngology, Head and Neck Surgery, Faculty of Medicine, Semmelweis University, 1085 Budapest, Hungary; tamas.laszlo@semmelweis.hu; 3 Department of Voice Speech and Swallowing Therapy, Faculty of Health Sciences, Semmelweis University, 1085 Budapest, Hungary

**Keywords:** odontogenic sinusitis, complex therapy in odontogenic sinusitis, chronic maxillary sinusitis, FESS

## Abstract

Objective: Odontogenic sinusitis (OS) is a well-known and important border of specialties in otorhinolaryngology and dentoalveolar surgery. Odontogenic sinusitis can develop due to iatrogenic harm or odontogenic infection. The gold standard diagnostic method is clinical and radiological—CBCT (cone beam computed tomography)—examination. The treatment of this condition requires collaboration between ENT and dentoalveolar surgery specialists and can be non-surgical or surgical based on staging. This paper aims to share the results of our clinical study whereby complex therapy was administered by a dentoalveolar surgeon and an otorhinolaryngologist in cooperation. Patients and methods: We conducted a retrospective study comprising 111 OS patients who underwent complex therapy between 2016 and 2023 at Semmelweis University, Budapest, Hungary. All patients were treated with concurrent FESS (functional endoscopic sinus surgery) and dentoalveolar surgery. Follow-up was based on symptoms, clinical examination and CBCT imaging. Results: Of the 111 patients, 107 were successfully treated with concurrent FESS and dentoalveolar surgery, and only 4 had further symptoms following the complex therapy and needed retreatment. Conclusions: The complex, single-session therapy involving FESS and oral surgery is an effective treatment method, which is less invasive and associated with fewer complications compared to previous interventions, such as the Luc–Caldwell procedure.

## 1. Introduction

### 1.1. Prevalence

Odontogenic sinusitis is the inflammation of the maxillary sinus which develops through the violation of the Schneiderian membrane [1]. According to most studies, odontogenic sinusitis accounts for 10–14% of all types of maxillary sinusitis [2,3,4]. However, some studies report a much higher incidence, at approximately 40% [5].

### 1.2. Causes of Odontogenic Sinusitis

**Figure 1:**
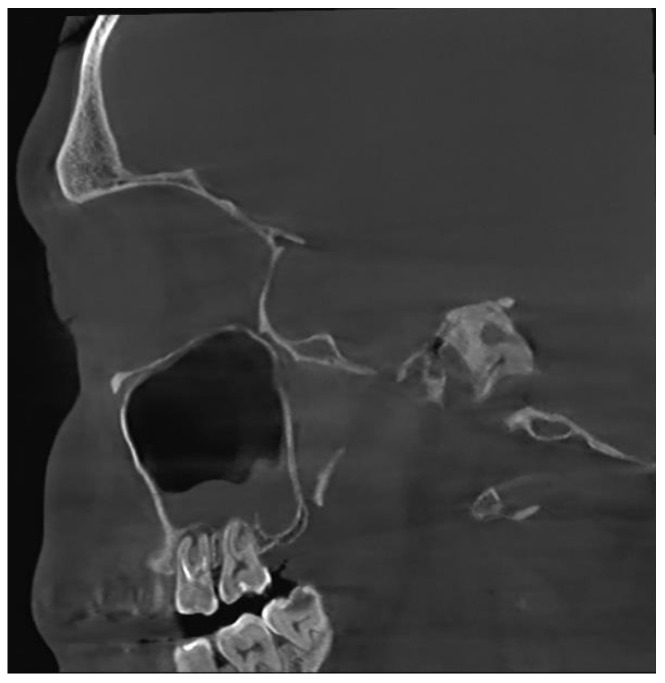
CBCT image of odontogenic sinusitis caused by iatrogenic harm via failed endodontic treatment: upper first and second molar periodontitis, chronic apical periodontitis (sagittal view).

Unfortunately, nowadays, the most common cause of odontogenic sinusitis is iatrogenic harm (approximately 56%), with most cases occurring in patients who underwent dental interventions. Iatrogenic harm occurs upon incorrectly performed sinus lift procedures or implant placement, foreign bodies, dental extractions with or without pushing a fragment of the root into the sinus cavity, orthognathic surgery, labio-palatine cleft surgery, Le Fort osteotomies, inadequate oro-antral fistula closure procedures, periimplantitis or endodontic treatment failure (Figure 1).

**Figure 2:**
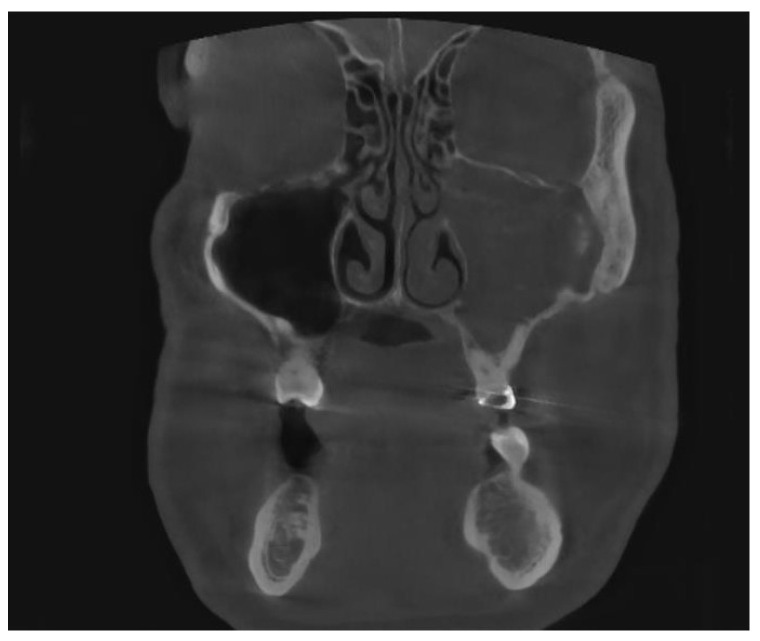
CBCT image of unilateral odontogenic maxillary sinusitis caused by periapical lesion. Maxillary sinus filled with secretions (coronal view).

Besides iatrogenic harm, odontogenic infections (dental caries, endodontic infections, periapical lesions (Figure 2), periodontal pockets, complex endo-periodontal lesions odontogenic cysts and traumatic injuries of the maxillary bone can also lead to odontogenic sinusitis [6].

### 1.3. Stages of Odontogenic Sinusitis

**Figure 3:**
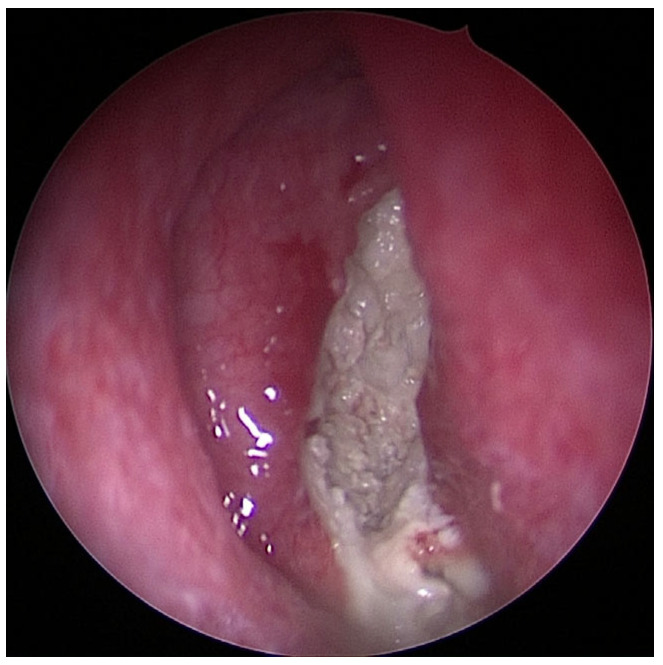
Image of chronic purulent odontogenic maxillary sinusitis with fungal superinfection via FESS (endoscope with straight–forward optic 0°).

In order to assess the severity of odontogenic maxillary sinusitis, it is important to have a clear understanding of the stages of inflammation progression. In the initial phase of inflammation, the inner mucous membrane lining the maxillary sinus (Schneiderian membrane) becomes affected around the alveolar recess. From there, it spreads circumferentially around the inner surface of the sinus, eventually reaching the infundibulum and closing off the natural opening. In cases of chronic inflammation, the maxillary sinus becomes filled with secretions. Following this, the inflammation spreads to anterior ethmoid cells. In some cases, inflammation can even extend to the other paranasal sinuses and the contralateral maxillary sinus [7]. If there is only swelling of the alveolar recess mucous membrane, performing FESS (Functional Endoscopic Sinus Surgery) is not justified. However, in the case of blockage of the natural opening—closed infundibulum—surgical therapy is recommended (Figure 3).

In clinical staging, acute (symptom duration less than 2 weeks), subacute (symptom duration between 4 and 12 weeks), recurrent acute sinusitis (four or more episodes of acute sinusitis per year without persistent symptoms between the episodes) and chronic odontogenic sinusitis (with a symptom duration longer than 12 weeks) can be distinguished [8].

### 1.4. Symptoms

In chronic inflammation, the most frequent symptom is purulent rhinorrhea. This symptom occurs in nearly 67% of patients [9]. Other typical symptoms include one-sided nasal congestion and foul-smelling taste or odor, which can be the consequence of a bacterial infection. Less commonly reported symptoms include toothache, facial pain, headache, sensitivity to pressure in the affected sinus area and postnasal drip, which can cause the patient to complain of coughing. None of these symptoms are specific to the origin of sinusitis. Some patients also mention dental complaints. Hypersensitivity and pain can occur in the upper teeth, especially in the first and second molars; however, this is not specific to OS [4].

Sometimes odontogenic sinusitis can develop without any alarming symptoms and radiological signs of inflammation are only discovered as an incidental finding on a CBCT (cone-beam computed tomography) scan—an appropriate diagnostic method for sinus evaluation—requested for another purpose by the treating physician [10,11].

### 1.5. Diagnostics

For a proper diagnosis, a complex, multi-step examination is required, beginning with a detailed medical history, followed by a clinical examination of symptoms. Predisposing factors of non-odontogenic sinusitis, such as allergies, asthma bronchiale and acetylsalicylic acid hypersensitivity, require special attention, and in cases of allergic patients, allergy testing (Prick test or specific IgE test) is essential. Asthma bronchiale needs special attention, as it is one of the most common predisposing factors of CRSwNP (chronic rhinosinusitis with nasal polyps). 

Clinical examination is important and includes examination of the oral cavity and teeth. Examination can reveal painful palpation of the anterior wall of the maxillary sinus, percussion sensitivity of the posterior teeth, necrosis of the dental pulp, presence of oroantral fistulas, existence of inflamed or fractured teeth and existence of dental implants, providing clues for localizing the lesion and causative dental unit [12]. During clinical examinations, anterior rhinoscopy and epipharyngoscopy have to be performed, highlighting changes in the mucosa and the presence of pus in the nostril or the middle meatus. During this examination, the operator can gather useful information about the patient’s unique anatomy, such as septum deviation, concha bullosa or narrow nasal meatus, which helps in planning the FESS procedure [13].

For proper diagnosis and treatment planning, CBCT is widely used as a “gold standard” method alongside clinical examination to confirm the anatomical landmarks and the diagnosis, to localize and measure the extensions of the pathologic lesions [14]. CBCT can produce detailed three-dimensional views of jaw bones, teeth and related pathologies. The evaluator can examine the sinus–dentoalveolar complex and maxilla in axial, sagittal and coronary views on the image, which offers more details and accuracy for the diagnosis and can help plan the endoscopic surgery. CBCT has a lot of advantages over classic CT, such as a 10% lower irradiation dose compared to CT, shorter examination time, more comfortable position, higher resolution and lower cost [15,16].

### 1.6. Treatment Options

Optimal treatment of odontogenic sinusitis calls for the collaboration between otorhinolaryngologists, dentoalveolar surgeons and dentists. The therapeutic algorithm usually includes two stages: conservative and surgical. The aim of the therapy is to eliminate the underlying cause, relieve symptoms and restore the normal function of the sinus [17]. During treatment, the dental infection source and inflammation of the sinus cavity have to be eliminated as well. Antibiotic treatment, endodontic therapy, nasal steroids and decongestants can be considered as conservative therapy [18,19,20]. 

If the underlying cause is inflammation in the periapical area, root canal treatment endodontic retreatment or endodontic microsurgery are possible therapies [18]. The success rate of initial endodontic therapy varies between 53 and 98%, as reported in studies conducted for the first attempt. However, the success rate is lower for cases requiring retreatment and involving periapical lesions. Numerous practitioners hesitate to perform endodontic surgery on posterior teeth due to potential infringement upon the maxillary sinus in the maxillary arch. However, these concerns should not discourage surgical interventions, as there are established techniques to effectively manage them. Perforating the sinus can happen quite easily when working with posterior teeth in the upper jaw. This can sometimes be prevented by carefully conducting the surgical procedure, but there are instances where it cannot be avoided, especially if the roots extend into the sinus. In case of a sinus perforation, the crucial step is to prevent any solid particles, such as cotton pellets or root-end filling materials, from entering the sinus cavity. The sinus has the capability to flush away fluids, but not solid substances [21]. Endodontic surgery can be contraindicated where the presence of delicate anatomical structure—such as the maxillary sinus—in close proximity could result in temporary or permanent damage during the surgical procedure. Targeted surgical approaches or guided endodontic microsurgery can be an appropriate solution in these challenging situations; however, these treatment possibilities also have their limitations [22]. 

The use of decongestants can be beneficial in cases where the patient complains about nasal congestion. Ephedrine is a well-known molecule in the treatment of nasal congestion due to its vasoconstriction action on the nasal mucosa. Ephedrine applied to the nasal mucosa reduces nasal resistance really quickly but at end of treatment, there may be a rebound effect with increased nasal resistance and recurrence of congestion can occur [23]. Nasal steroids are suggested in allergic rhinitis and chronic rhinosinusitis as well, for the purpose of reducing mucosal swelling and oedema. These drugs are often used in combination with antibiotics in odontogenic sinusitis [19]. 

At least 10% of acute sinusitis cases have a dental origin. According to some studies 60% of acute sinusitis cases can heal spontaneously without antibiotics. However, in some cases, antibiotics may be necessary; targeted therapy should be applied based on antibiotic sensitivity testing whenever possible. Empirical therapy is often used for acute suppurative sinusitis, based on clinical symptoms. Odontogenic sinusitis is associated with anaerobic bacterial flora. The first-line treatment options are broad-spectrum penicillins (aminopenicillins). Aminopenicillins are often combined with β-lactamase inhibitors (e.g., 875 mg amoxicillin and 125 mg clavulanic acid). As an alternative, second-generation oral cephalosporins (cefuroxime axetil) can be used. The spectrum of second generation cephalosporins are similar to aminopenicillin combined with beta-lactamase inhibitor, but they are less effective against anaerobic bacteria, making them less advantageous in the case of odontogenic sinusitis. Patients with a penicillin allergy should be treated with a fixed-dosed trimethoprim/sulfamethoxazole combination. Cephalosporins should be avoided due to cross-allergies. In case of unsuccessful antibiotic treatment, the used drug must be changed to another one and antibiotic combination should be applied. Intravenous administration is justified only in severe cases, where the risk of inflammation spreading to the intracranial or orbital area is high. For targeted antibiotic therapy, the patient needs sampling, bacterial culture and antibiotic sensitivity testing. Targeted therapy prevents the spread of resistant strains and makes the treatment more effective [24].

During conservative therapy, besides medical treatment, any necessary dental treatments identified during the oral examination need to be carried out, not limited to treating only the tooth causing odontogenic sinusitis. Dental hygiene treatment and customized oral hygiene education, treatment of dental pockets, root canal treatment, caries removal, tooth extraction must be performed if necessary. This step is important before surgical therapy as well. With an organized dental status, the healing is more favorable.

**Figure 4:**
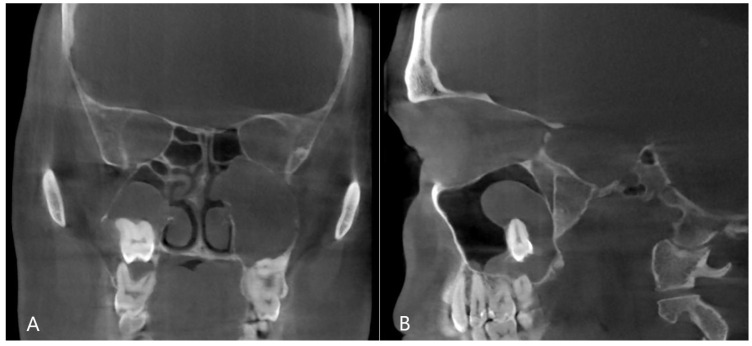
CBCT image of odontogenic cyst with calcified capsule, highly impacted upper wisdom tooth in the right maxillary sinus. Left chronic odontogenic sinusitis caused by impacted left upper wisdom. ((**A**): coronal, (**B**): sagittal view).

Among the results of studies examining the effectiveness of conservative therapy, there is a significant variation, with the results ranging between 36% and 95% [12]. After a period of six weeks, a follow-up examination should be conducted to assess the healing progress of the odontogenic sinusitis. If there are no signs of healing or if the symptoms persist, invasive therapy may be required. In some cases of odontogenic sinusitis, the inflammation can progress to the extent that the natural opening of the maxillary sinus—the infundibulum—becomes completely blocked. In such cases, improvement cannot be expected through conservative therapy and surgical intervention is usually recommended (Figure 4).

**Figure 5:**
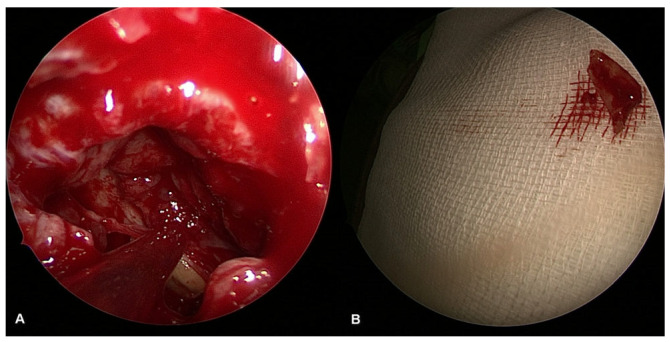
Surgical image of radix removal from the sinus cavity ((**A**): radix inside the maxillary sinus, (**B**): removed radix).

The first-line surgical treatment should be a combined FESS and oral surgery procedure such as tooth extraction, oro-antral fistula closure and foreign body removal (Figure 5), performed in a single session. The less commonly used procedures today are the Lothrop, Denker and Luc–Caldwell surgeries. Studies examining the effectiveness of simultaneous FESS and dentoalveolar surgery report better results compared to conservative therapy. According to most studies, the effectiveness of this treatment is reported to be between 95% and 100% after unsuccessful conservative therapy. FESS is a functional endoscopic sinus surgery based on a functional approach. The aim of this endoscopic procedure is to restore ventilation of the paranasal sinuses [6,12].

The most common indication for FESS is chronic rhinosinusitis (CRS). Surgical indication is only considered when conservative therapy has failed. Other indications include chronic odontogenic sinusitis, complications of rhinosinusitis (intraorbital and intracranial complications), benign tumors of the paranasal sinuses, dacryocystorhinostomy (DCR), transnasal hypophysectomy, closure of liquor fistulas, orbital decompression, orbital tumors, trauma cases, management of nose bleeding, selected cases of malignant tumors, such as juvenile nasopharyngeal angiofibroma (JNA), and tumors in the pterygopalatinal and infratemporal fossa [25,26].

Sinus ventilation and restoration of mucociliary clearance can be ensured by widening the natural meatus of the maxillary sinus. Depending on the size of the surgical area, at least 6 weeks postoperative therapy is required to restore mucociliary transport [27].

The potential complications of the procedure fall into two broad categories. These are minor and major complications [26]. Minor complications may occur more frequently, but if detected in time, they can be well treated, even during the surgery. Major complications are rare. It is crucial to detect and treat these serious complications as soon as possible, as they can be fatal if left untreated. 

Minor complications include synechiae, hemorrhages or damage of the nasolacrimal duct, which can cause tearing or permanent tear duct obstruction, double vision due to injury to the musculus rectus medialis, orbital emphysema caused by damage to the lamina papyracea and loss of sense of smell.

Major complications include severe injuries that can affect the anterior and posterior ethmoid arteries, the internal carotid artery (which can cause life-threatening bleeding), the optic nerve, the orbit and orbital contents (retrobulbar hematoma, periorbital emphysema) and the dura. Cerebrospinal fluid leak and other intracranial complications may also occur. 

It is important to emphasize that these complications rarely occur and can be prevented with correct indication, appropriate radiological imaging and thorough preparation by the surgeon. Due to technological advances and improved surgical training, the outcomes of FESS have significantly improved in recent years [25,26,28].

In addition to considering all these aspects, practical experience shows that in odontogenic maxillary sinusitis of any severity, expanding the natural opening and middle nasal passage, as well as performing sinus lavage enhances the prospects of recovery. In certain cases, septoplasty is also performed to ensure proper sinus ventilation [12].

## 2. Materials and Methods

We analyzed the data of 111 patients with odontogenic sinusitis who underwent complex therapy between 2016 and 2023 at Semmelweis University, Budapest, Department of Otorhinolaryngology, Head and Neck Surgery in cooperation with the Department of Oral Diagnostics. The data were acquired from the MedSol Database and analyzed retrospectively based on demographic and anamnestic data, as well as clinical symptoms.

All patients were asked in detail about their anamnestic data and medical history, highlighting their complaints and previous treatments. Patients with possible allergies went through allergy testing (prick test or specific IgE determination). Alongside dental and general examination, a complete otorhinolaryngological examination was performed by an otorhinolaryngologist. Anterior rhinoscopy and epipharyngoscopy was crucial for all patients undergoing FESS.

The inclusion criteria were simultaneous sinusitis and a dental infection source. Sinusitis had to be confirmed via otorhinolaryngological examination (image of purulent sinusitis via rhinoscopy, purulent discharge from the middle meatus) and/or CBCT imaging (involvement of maxillary sinus, swelling and oedema of the alveolar recess and sinus filled with secretions) and symptoms. Tooth-related inflammation or iatrogenic harm (periodontitis, endodontic infections, periapical lesions, foreign bodies, oro-antral fistulas, failed implant therapy, odontogenic cysts) had to be confirmed via clinical examination by maxillofacial surgeon and radiological imaging (CBCT). Patients who had sinusitis without proven tooth inflammation or iatrogenic harm, and patients with CRSwNP (chronic rhinosinusitis with nasal polyps) were excluded.

Before the intervention, nasal steroid spray (Mometasone furoate 2 × 100 μg per day) was administered locally, and nasal saline irrigation was suggested 3 times a day for 2 to 3 weeks before the surgery for all patients. If there was no penicillin allergy, 1000 mg amoxicillin and 200 mg clavulanic acid was administered parenteral perioperative. Patients with a penicillin allergy were given 600 mg clindamycin. Antibiotics were necessary due to oral surgery; however, preoperative administration of antibiotics is not recommended for non-odontogenic sinusitis cases [29]. 

The operation was performed by an otorhinolaryngologist and a dentoalveolar surgeon in every case. A sterile gauze, soaked in an Etamsylat–Ephedrine–Marcaine solution was placed into the operated nostril for 5 min. Following this, local anesthetic infiltration of lidocaine 1% with adrenalin 1:200,000 solution for injection was performed in the middle turbinate, the origin of the turbinate and the lateral nasal wall. The next step was infundibulotomy, ensuring complete removal of the uncinate process. The maxillary sinus ostium (using Stammberger maxillary sinus dilatator) was dilatated. Initially, a 0-degree endoscope was used, but for clearing the maxillary sinus, we switched to a 30- or 45-degree endoscope. After cyst removal or suctioning the purulent discharge, the cavity was irrigated with normal saline or 1–2% H_2_O_2_ solution. If necessary, we cleared the ethmoid cells or, in rare cases involving the frontal and sphenoid sinus, dilated their ostia, suctioned and irrigated. Finally, hemostasis was achieved and a sterile gauze strip was inserted into the patient’s nostril for a maximum of 24 h, for the purpose of nasal packaging, thereby decreasing the chance of postoperative bleeding and preventing lateralization of the middle turbinate into the infundibulum [30]. During complex therapy, a dentoalveolar surgeon performed the required oral surgery procedures in addition to FESS. 

All patients went through dentoalveolar intervention alongside FESS surgery. The most frequently needed procedures were tooth extraction, oro-antral fistula closure and cystectomy. For the closure, we used a buccal flap (Wasmund) to close the oro-antral fistula in most cases. After excising the epithelialized margins, two vertical release incisions were made to develop a flap with adequate dimensions for closure of the defect. The epithelial lining of the fistula was also removed. This trapezoidal flap consists of both the epithelium and connective tissue. The flap is then dissected and stretched as needed, placed over the defect and sutured in place by a non-absorbable 4.0 suture. In cases where there is a need for tooth extraction or surgical removal of the teeth, it is performed after the full thickness muco-periostal flap is made. After 10 days, sutures are removed by the surgeon. 

Postoperative antibiotic treatment was administered (875 mg amoxicillin/125 mg clavulanic acid twice a day for 7 days was the first-line option, and, for patients with a penicillin allergy, clindamycin 300 mg four times daily for 7 days was prescribed). Postoperative antibiotics were necessary due to OAF closure and dentoalveolar surgery. Nasal steroids (Mometasone furoate 2 × 100 μg/day), nasal saline irrigation 3 times/day, COLDASTOP^®^ 15,000 NE/mL retinol + 20 mg/mL tocopherole 3 times 2–3 drops/day were suggested for 2 to 3 weeks after surgery. This combination of local intranasal therapy promotes better and faster regeneration of the nasal mucosa. Follow-up included clinical examination and CBCT imaging 6 months after surgery. 

## 3. Results

The results are shown in Table 1. Of the 111 patients diagnosed with odontogenic sinusitis, 56 were female (50.5%) and 55 were male (49.5%), with an average age of 45 years. Of the patients, 26% (*n* = 28) were between 31 and 40 years old.

**Figure 6:**
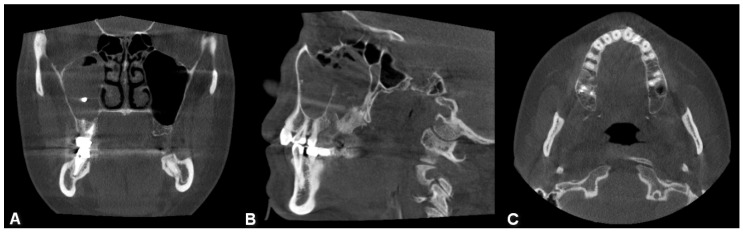
CBCT image of unilateral maxillary sinusitis and oro-antral fistula with the characteristic radiological image of fungal infection, which radiologically mimics foreign bodies ((**A**): coronal, (**B**): axial, (**C**): sagittal view).

An average of 9 months passed between the onset of symptoms and surgery. Of the patients, 32% were asymptomatic (*n* = 36). The most common complaint was nasal discharge, which occurred in 24% of patients (*n* = 27); 14% complained of tooth pain (*n* = 16) and nasal congestion; and 13% reported postnasal drip (*n* = 14) and facial pain. Fewer patients experienced the following symptoms: recurrent sinusitis, ear problems, oro-antral fistula (Figure 6), cough, facial swelling in the maxillary sinus area, decreased sense of smell, snoring, nasal bleeding and strange taste sensation.

The following reasons were behind the development of odontogenic sinusitis: Of the 111 patients, there were 43 periapical lesions (39%), 26 oro-antral fistulae after extraction (23%), 11 cases of periodontal lesion (10%), 9 cysts (8%), 9 endodontic treatment failure (8%), 7 foreign body/implant placement (6%), 5 impacted wisdom teeth (5%) and 1 supernumerary tooth (0.9%).

Of the 111 patients, 84 cases (76%) were unilateral and 27 (24%) were bilateral odontogenic sinusitis. Pansinusitis was observed in 30 cases (27%), while in 79 cases (71%), only the maxillary sinus was involved, and, in 2 cases (2%), both the maxillary and ethmoid sinuses were affected. Of the patients, 19% smoked, 78% were self-reported non-smokers and 3% had quit smoking.

In 89 cases (80%), patients did not report any predisposing factors, 15 patients were allergic (14%), 2 patients received bisphosphonate treatment (2%) and polypoid lesions were observed in 4 patients (4%) on CT scans. 

**Figure 7:**
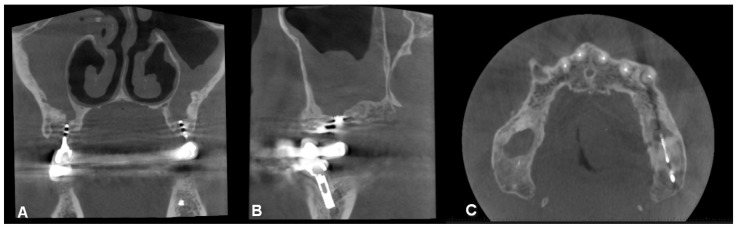
CBCT image of bilateral maxillary sinusitis caused by blade implants; chronic OAF (oroantral fistula) next to the implants. ((**A**): coronal, (**B**): sagittal, (**C**): axial view).

Among the patients, 63 (58%) did not have previous dental treatment performed by other physicians before the development of OS, while 48 (42%) mentioned previous dental intervention. Extraction was the most common dental procedure (27%), followed by root canal treatment in six patients (5%), sinus closure in three patients (3%), implant placement (Figure 7) and root apex resection in two patients each (2%).

Some patients underwent multiple procedures before the onset of inflammation. Among the patients, 86 (77%) did not receive prior treatment. FESS was performed in nine cases, punction and lavage in two cases, Luc–Caldwell operation in two cases, and five patients underwent conservative antibiotic therapy. Oro-antral fistula closure was performed in two patients, and one patient previously had FESS + Luc–Caldwell, septum correction, Lothrop surgery, cyst extraction and septoplasty.

The therapy was always complex. In addition to FESS, fistula closure was indicated in 70 patients (63%), extraction in 64 patients (58%), foreign body removal in 4 patients (4%) (Figure 5), cystectomy in 7 patients (6%) and septoplasty in 10 patients (9%). Some patients required multiple dental interventions in addition to FESS surgery.

## 4. Discussion

Odontogenic sinusitis is a very common and underestimated disorder, which deserves more attention. Among the patients examined in this study, recurrence occurred in four patients (3.6%), resulting in a success rate of 96.4%. The low recurrence rate, good healing tendency, and few postoperative complications lets us suggest that this procedure is an effective, up-to-date method for the treatment of odontogenic sinusitis, in such cases where conservative therapy was unsuccessful.

We compared our results with the data found in the international literature on single-step surgical treatment. The majority of case series reported about 90–100% success rate [12]. To the authors’ knowledge, there are only a few case series with long-term follow-up on a high number of patients. Felisati et al. (2013) reported that only 3 of 257 patients required a second surgical procedure, with the mean follow-up of 25 months [31]. Costa et al. (2019) reported 93% resolution based on symptoms, endoscopy and sinus CT or CBCT. The number of patients was 88 in this study, with a 6-month follow-up [12,32].

Use of the previously routine Luc–Caldwell surgery is decreasing due to the development of endoscopic techniques and the occurrence of serious complications. In a study focusing on multidetector-computed tomography imaging, several patients who underwent the Luc–Caldwell procedure were examined. Overall, in 92.9% of patients, imaging confirmed sinonasal complications. The following complications were observed: anterior and medial osseus wall defect, collapse of the sinus cavity, sclerosis and sinus wall thickening. Beside these complications, severe bleeding may occur if the sphenopalatine artery is injured in the fossa infratemporalis or fossa pterygopalatina during the surgery. After healing, the physiological ciliated mucosa layer is replaced by a squamous epithelium, causing dysfunction. Due to the extensive exposure, sensory disturbances may occur in the upper teeth, and patients often report postoperative numbness and facial swelling [33].

These data highlight that further investigations are needed involving detailed symptoms, anamnestic data, predisposing factors, perioperative, postoperative complications and long-term follow-ups. Although the use of FESS and simultaneous dentoalveolar procedures in OS is a well-known method to treat the inflamed maxillary sinus, there is still a lack of studies with cases reporting numerous patients and long-term follow-up [34].

## 5. Conclusions

**Figure 8:**
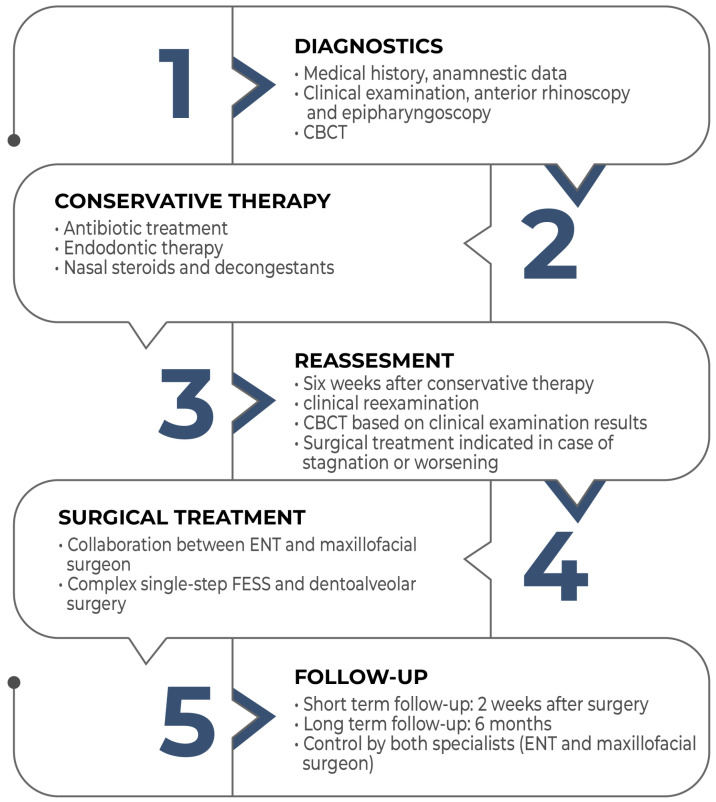
Flowchart of the management of odontogenic sinusitis.

Despite the relatively high frequency of odontogenic sinusitis, its significance is underestimated. Its treatment always requires collaboration between an ENT specialist and a dentoalveolar surgeon. Complex, single-session therapy involving FESS and oral surgery is an effective treatment method. However, to establish the widespread adoption of this complex, single-session therapy, further studies on a large number of patients with long-term follow-ups and the development of protocols—as shown in the flowchart below- would be necessary (Figure 8).

## Figures and Tables

**Table 1:** Demographic data and results of 111 patients treated with concurrent FESS and dentoalveolar surgery.

**Demographic data**		**Most common symptoms**		**Cause of the OS**	
Gender	50.5% female 49.5% male	Asymptomatic	32%	Periapical lesions	39%
		Nasal discharge	24%	Oro-antral fistula after extraction	23%
		Tooth pain	14%	Periodontal lesion	10%
		Nasal congestion	14%	Cysts	8%
Average age	45 years	Postnasal drip	13%	Endodontic treatment failure	8%
		Facial pain	13%	Foreign body/implant placement	6%
				Impacted wisdom teeth	5%
**Sinus involvement**		**Previous dental treatment**		**Previous treatment**	
Unilateral	76%	Had no intervention	58%	No previous treatment	77%
Bilateral	24%	Extraction	27%	FESS	8%
		Root canal treatment	5%	Antibiotic treatment	5%
Sinus maxillaris	71%	Sinus closure	3%	Punction and lavage	2%
Pansinusitis	27%	Implant placement	2%	Luc–Caldwell	2%
Sinus maxillaris and ethmoidalis	2%	Root apex resection	2%	Other treatment	6%
**Smoking habits**		**Predisposing factors**		**Additional therapies besides FESS**	
Non-smoker	78%	None	80%	Fistula closure	63%
		Allergies	14%	Extraction	58%
Active smoking	19%	Polypoid lesions	4%	Septoplasty	9%
		Bisphosphonate	2%	Cystectomy	6%
Ex-smoker	3%			Foreign body removal	4%

## Data Availability

Available on request.

## References

[1] Newsome H.A., Poetker D.M. (2020). Odontogenic Sinusitis: Current Concepts in Diagnosis and Treatment. Immunol. Allergy Clin. N. Am..

[2] Longhini A.B., Branstetter B.F., Ferguson B.J. (2010). Unrecognized Odontogenic Maxillary Sinusitis: A Cause of Endoscopic Sinus Surgery Failure. Am. J. Rhinol. Allergy.

[3] Mehra P., Jeong D. (2009). Maxillary sinusitis of odontogenic origin. Curr. Allergy Asthma Rep..

[4] Brook I. (2006). Sinusitis of odontogenic origin. Otolaryngol. Head Neck Surg..

[5] Patel N.A., Ferguson B.J. (2012). Odontogenic sinusitis: An ancient but under-appreciated cause of maxillary sinusitis. Curr. Opin. Otolaryngol. Head Neck Surg..

[6] Martu C., Martu M.A., Maftei G.A., Diaconu-Popa D.A., Radulescu L. (2022). Odontogenic Sinusitis: From Diagnosis to Treatment Possibilities-A Narrative Review of Recent Data. Diagnostics.

[7] Psillas G., Papaioannou D., Petsali S., Dimas G.G., Constantinidis J. (2021). Odontogenic maxillary sinusitis: A comprehensive review. J. Dent. Sci..

[8] Rosenfeld R.M., Andes D., Bhattacharyya N., Cheung D., Eisenberg S., Ganiats T.G., Gelzer A., Hamilos D., Haydon R.C., Hudgins P.A. (2007). Clinical practice guideline: Adult sinusitis. Otolaryngol. Head Neck Surg..

[9] Shanbhag S., Karnik P., Shirke P., Shanbhag V. (2013). Association between periapical lesions and maxillary sinus mucosal thickening: A retrospective cone-beam computed tomographic study. J. Endod..

[10] Dumitrescu D., FănuŢă B., Stepan A.E., Fronie A.I., Dumitrescu C.I., MârŢu M.C., Şurlin P., Şurlin V., Popescu M. (2015). Silent sinus syndrome—Report of a case. Rom. J. Morphol. Embryol..

[11] Bisla S., Gupta A., Singh H., Sehrawat A., Shukla S. (2022). Evaluation of relationship between odontogenic infections and maxillary sinus changes: A Cone Beam Computed Tomography-based study. J. Oral Biol. Craniofacial Res..

[12] Craig J.R., Tataryn R.W., Aghaloo T.L., Pokorny A.T., Gray S.T., Mattos J.L., Poetker D.M. (2020). Management of odontogenic sinusitis: Multidisciplinary consensus statement. Int. Forum Allergy Rhinol..

[13] Nurchis M.C., Pascucci D., Lopez M.A., Moffa A., Passarelli P.C., Bressi F., Casale M., Damiani G. (2020). Epidemiology of odontogenic sinusitis: An old, underestimated disease, even today. A narrative literature review. J. Biol. Regul. Homeost. Agents.

[14] Szabo B.T., Aksoy S., Repassy G., Csomo K., Dobo-Nagy C., Orhan K. (2017). Comparison of hand and semiautomatic tracing methods for creating maxillofacial artificial organs using sequences of computed tomography (CT) and cone beam computed tomography (CBCT) images. Int. J. Artif. Organs.

[15] Molteni R. (2021). The way we were (and how we got here): Fifty years of technology changes in dental and maxillofacial radiology. Dentomaxillofac. Radiol..

[16] Antohi C., Salceanu M., Aminov L., Martu M.-A., Dascalu C.G., Dodi G., Stoica G., Bandol G., Iancu D., Dobrovat B. (2022). Assessment of Systemic and Maxillary Bone Loss in Cancer Patients with Endo-Periodontal Lesions Using Dkk-1 Biomarker and Dental Radiological Examinations. Appl. Sci..

[17] Saibene A.M., Pipolo C., Borloni R., Felisati G. (2021). ENT and dentist cooperation in the management of odontogenic sinusitis. A review. Acta Otorhinolaryngol. Ital..

[18] Hauman C.H.J., Chandler N.P., Tong D.C. (2002). Endodontic implications of the maxillary sinus: A review. Int. Endod. J..

[19] Pedlar J., Frame J.W. (2007). Oral and Maxillofacial Surgery: An Objective-Based Textbook.

[20] Dhingra P., Dhingra S. (2018). Diseases of Ear, Nose and Throat & Head and Neck Surgery.

[21] Kim S., Kratchman S. (2006). Modern endodontic surgery concepts and practice: A review. J. Endod..

[22] Setzer F.C., Kratchman S.I. (2022). Present status and future directions: Surgical endodontics. Int. Endod. J..

[23] Laccourreye O., Werner A., Giroud J.P., Couloigner V., Bonfils P., Bondon-Guitton E. (2015). Benefits, limits and danger of ephedrine and pseudoephedrine as nasal decongestants. Eur. Ann. Otorhinolaryngol. Head Neck Dis..

[24] Katzung B.G., Vanderah T.W. (2021). Basic & Clinical Pharmacology.

[25] Homsi M.T., Gaffey M.M. (2023). Sinus Endoscopic Surgery. StatPearls.

[26] Sireci F., Lorusso F., Martines F., Salvago P., Immordino A., Dispenza F., Gallina S., Canevari F.R. (2021). Guide to the management of complications in endoscopic sinus surgery (ESS). Adv. Health Dis..

[27] Aroor R., Sunu Ali Z., Gangadhara Somayaji K.S. (2017). Do Nasal Surgeries Affect Mucociliary Clearance?. Indian J. Otolaryngol. Head Neck Surg..

[28] Krings J.G., Kallogjeri D., Wineland A., Nepple K.G., Piccirillo J.F., Getz A.E. (2014). Complications of primary and revision functional endoscopic sinus surgery for chronic rhinosinusitis. Laryngoscope.

[29] Lehmann A.E., Raquib A.R., Siddiqi S.H., Meier J., Durand M.L., Gray S.T., Holbrook E.H. (2021). Prophylactic antibiotics after endoscopic sinus surgery for chronic rhinosinusitis: A randomized, double-blind, placebo-controlled noninferiority clinical trial. Int. Forum Allergy Rhinol..

[30] Francesco L., Francesco D., Federico S., Michele M.D., Salvatore G. (2021). A Comparative Double Blind Study of Nasal Dressing Sponge® versus Merocel® as Nasal Pack after Nasal Surgery. Iran. J. Otorhinolaryngol..

[31] Felisati G., Chiapasco M., Lozza P., Saibene A.M., Pipolo C., Zaniboni M., Biglioli F., Borloni R. (2013). Sinonasal complications resulting from dental treatment: Outcome-oriented proposal of classification and surgical protocol. Am. J. Rhinol. Allergy.

[32] Costa F., Emanuelli E., Franz L., Tel A., Robiony M. (2019). Single-step surgical treatment of odontogenic maxillary sinusitis: A retrospective study of 98 cases. J. Craniomaxillofac. Surg..

[33] Schneider J.S., Day A., Clavenna M., Russell P.T., Duncavage J. (2015). Early Practice: External Sinus Surgery and Procedures and Complications. Otolaryngol. Clin. N. Am..

[34] Aukštakalnis R., Simonavičiūtė R., Simuntis R. (2018). Treatment options for odontogenic maxillary sinusitis: A review. Stomatologija.

